# Patent hepatic ciliated foregut remnant resulting in an umbilicobiliary sinus tract, with gallbladder agenesis, in an 8-wk-old male French Bulldog

**DOI:** 10.1177/10406387221147317

**Published:** 2023-01-04

**Authors:** Hannah E. Wong, John M. Cullen, Marta Vilà-González, Rachel Pittaway, Valeer J. Desmet, Tammy D. Gillian

**Affiliations:** Dick White Referrals, Six Mile Bottom, Cambridgeshire, UK; College of Veterinary Medicine, North Carolina State University, Raleigh, NC, USA; Cambridge Stem Cell Institute, University of Cambridge, Jeffrey Cheah Biomedical Centre, Cambridge Biomedical Campus, Puddicombe Way, Cambridge, UK; Dick White Referrals, Six Mile Bottom, Cambridgeshire, UK; Department of Pathology, Catholic University of Leuven, Leuven, Belgium; Oakwood Veterinary Referrals, Willows Veterinary Hospital, Hartford, Northwick, Cheshire, UK

**Keywords:** agenesis, cilia, cysts, dogs, embryonic structures, foregut remnant, fluorescent antibody technique, gallbladder, liver

## Abstract

Hepatic ciliated foregut remnants or cysts are congenital abnormalities resulting from retention of embryonic ciliated foregut within the liver. These structures are rarely reported in the human medical literature and have not been reported in the veterinary literature previously, to our knowledge. We describe here a case of an 8-wk-old male French Bulldog with a congenital patent hepatic ciliated foregut remnant resulting in an umbilicobiliary sinus tract. The dog also had concurrent gallbladder agenesis. The patient had yellow fluid discharging from the umbilicus, mimicking a patent urachus. Surgical exploration, removal, and histology provided a conclusive diagnosis of a hepatic foregut remnant and therapeutic resolution of the clinical signs. The histologic appearance of a hepatic foregut remnant is classical, namely a duct composed of 4 layers: an inner ciliated epithelial lining, loose connective tissue, smooth muscle, and a fibrous capsule.

An 8-wk-old male entire French Bulldog underwent investigation of yellow fluid discharging from the umbilicus. On initial examination, a patent urachus was suspected, and the patient was referred for surgical repair. At surgical consultation, it was documented that the dog urinated normally, and the liquid from the umbilicus had the appearance suggestive of bile, with yellow staining of the surrounding skin. The animal was otherwise clinically normal, with no steatorrhea.

At surgical exploration, an elliptical surgical incision was made around the umbilicus. A 24-G IV catheter (Smiths Medical International) was passed into the umbilical stoma and revealed a tract that extended along the medial aspect of the visceral surface of the right medial liver lobe (Fig. 1), connecting the umbilicus to the common bile duct. A gallbladder was not observed. The liver lobes, urinary bladder, and remaining abdominal organs were macroscopically normal. The urinary bladder had no association with the umbilical sinus tract. The tract and a small portion of the attached liver were excised with a small-jaw open sealer/divider (LigaSure; Covidien) and submitted for histology. The abdominal cavity was closed routinely, and the patient recovered uneventfully.

**Figures 1, 2.**
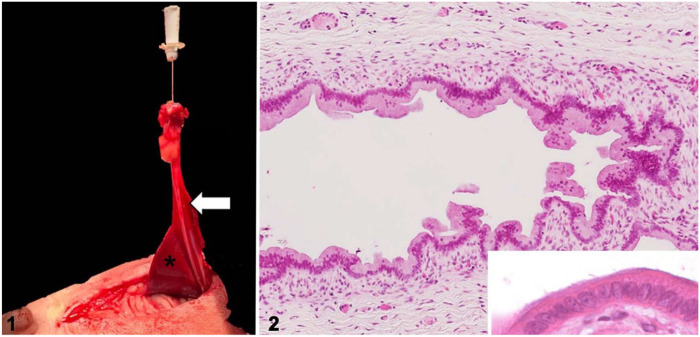
Hepatic foregut remnant in the liver of a dog. **Figure 1.** Exteriorization of the hepatic foregut remnant (arrow), highlighted by insertion of an IV catheter, as it extends along the medial aspect of the visceral surface of the right medial liver lobe (asterisk). **Figure 2.** Hepatic foregut remnant in the periumbilical region, composed of ciliated columnar epithelium supported by lamina propria and variable amounts of smooth muscle, which blend into a fibrous capsule. Inset: higher magnification of the ciliated epithelium. H&E.

Analysis of the dog’s blood biochemistry 1 wk post-surgery showed elevations in activities of alkaline phosphatase (548 IU/L; RI: 7–173 IU/L) and alanine transaminase (55 IU/L; RI: 0–40 IU/L). Aspartate aminotransferase, gamma-glutamyl transferase, glutamate dehydrogenase, bilirubin, and fasting bile acids were all within the appropriate RIs. At the time of writing, the dog was 16-mo-old. The animal remained clinically well, was considered normal in size, and had no biochemical abnormalities. Verbal informed consent from the owner of the dog was obtained for publication of this case.

The surgically excised tissue, a 50-mm long sinus tract bridging between the umbilicus and liver, was submitted for routine processing. Histologically, the sinus tract was a tubular structure lined by columnar ciliated epithelium supported by a lamina propria of loosely arranged spindloid cells and thin collagen bundles (Fig. 2). Folds in the ciliated epithelium formed glandular-type structures within the fibrous lamina propria. The epithelium and lamina propria were supported by a thin, incomplete, smooth muscle layer and a fibrous capsule (Suppl. Fig. 1). The lamina propria was infiltrated by low numbers of neutrophils, lymphocytes, and plasma cells. Immunofluorescence using anti–α-tubulin antibody highlighted cilia arranged in low-density aggregates on epithelial cells of the sinus tract (Figs. 3, 4; Suppl. immunofluorescence materials and method). The epithelial cells demonstrated nuclear FoxA2 immunofluorescence, consistent with foregut origin.

**Figure 3.**
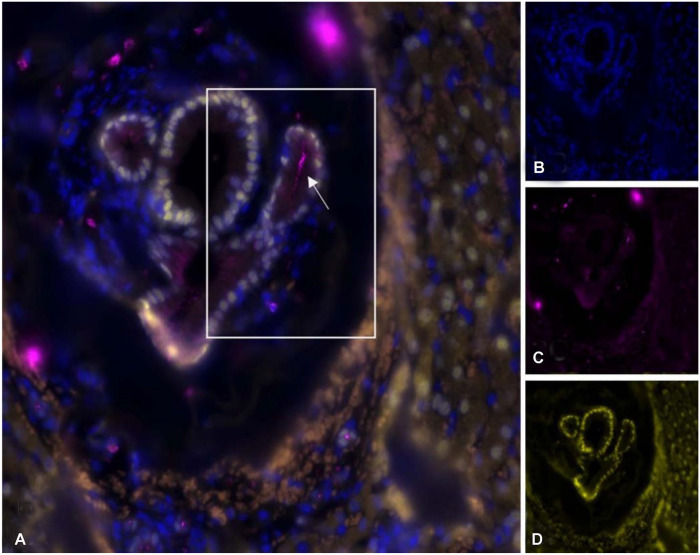
Immunofluorescent images of the hepatic foregut remnant in the liver of a dog. Pink = anti–α-tubulin antibody (ciliated epithelium, arrow), blue = 4′,6-diamidino-2-phenylindole (DAPI)-stained nuclei, yellow = anti-FOXa2 antibody. **A.** Perihepatic aspect of the ciliated foregut remnant. Composite image, **B–D.** Individual channels.

**Figure 4.**
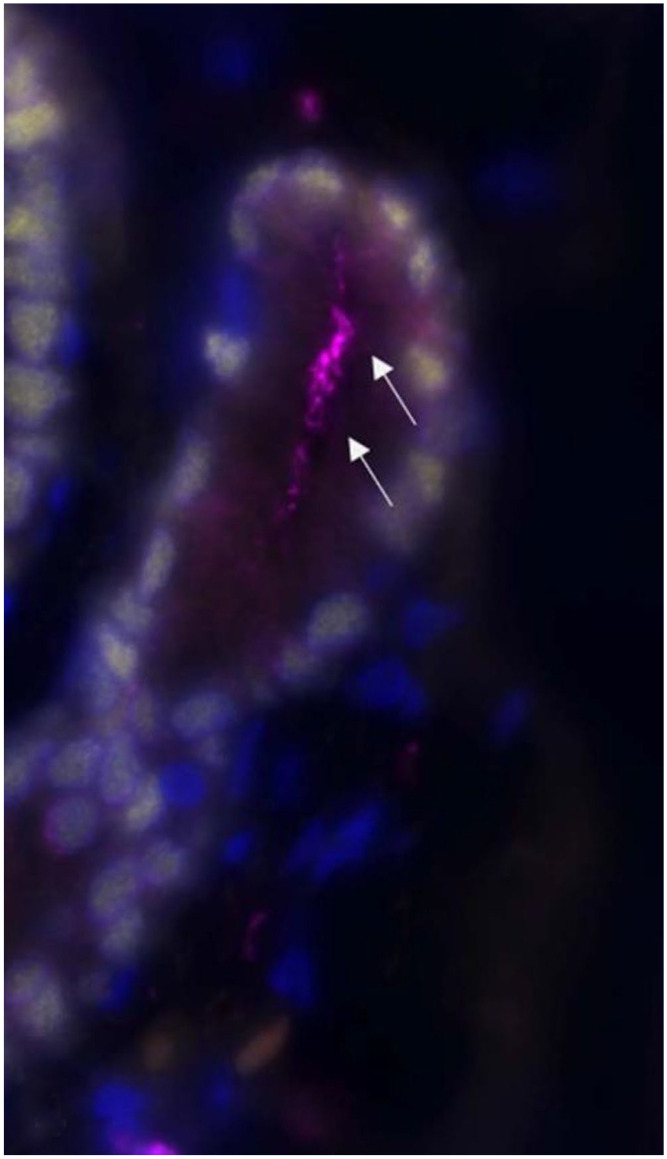
Magnification of box in Fig. 3A of the perihepatic aspect of the ciliated foregut remnant in the liver of a dog. Ciliated epithelium (arrows).

The perihepatic sinus tract, accompanied by large vessels and nerves, was supported by abundant reticulin fibers separated by RBCs (Suppl. Fig. 2). Adjacent to the tract, hepatic lobular architecture was present, but portal areas were abnormal. Smaller portal areas often lacked bile ducts, and larger portal areas lacked vessels but had biliary reduplication and portal fibrosis. The hepatic parenchyma contained randomly distributed small arterioles (Suppl. Fig. 3). Hemosiderin was not visible in hepatocytes or Kupffer cells in Perls Prussian blue–stained sections, suggesting that long-term congestion was not a sequela of the abnormal tissue architecture.

A patent connection between the common bile duct and the umbilicus by a duct lined with ciliated epithelium is consistent with a diagnosis of a hepatic foregut remnant. The lesion in our case demonstrated the classic histologic pattern involving 4 layers, consisting of an inner ciliated epithelial lining, loose connective tissue, smooth muscle, and a fibrous capsule.^2^ Cystic structures lined by ciliated epithelium in the region of the liver, derived from embryonic foregut, have been reported in humans.^1,6^ The embryonic anterior foregut normally gives rise to biliary, liver, pancreatic, proximal duodenum, stomach, lung, and esophageal tissues. The liver, trachea, and airways form from the ventral anterior foregut, and the esophagus forms from the dorsal anterior foregut.^7^ There are reported histologic similarities between ciliated hepatic foregut cysts and bronchial and esophageal cysts, and the cysts in our case reflect that common development.^20^

Other embryologic remnants that may communicate with the abdominal wall include a patent urachus and persistent portions of the vitelline (omphalomesenteric) duct. A patent urachus connects the urinary bladder and umbilicus and is lined by urothelium. The vitelline duct connects the yolk sac to the developing intestine in the embryo, but normally involutes in utero to form a residual fibrous cord. Failure of this involution may result in Meckel diverticulum, a blind-ending pouch that communicates with the intestinal lumen. Meckel diverticulum is relatively common in humans, horses, and pigs, but is rare in dogs.^19^ The central portion of the vitelline duct may fail to involute and result in cysts, lined by goblet cells and absorptive columnar cells, that do not communicate with the intestinal lumen.^5^ Other cystic structures arising in this area include biliary cyst, parasitic cyst, simple hepatic cyst, biliary cystadenoma, cystic metastasis, pancreatic pseudocyst, and gallbladder duplication. In our case, the identification of ciliated epithelium supported by loose connective tissue, smooth muscle, and a fibrous capsule, forming a luminal structure associated with the liver and biliary tree is diagnostic of a hepatic ciliated foregut cyst.^20^

Cilial morphology was visualized using immunofluorescence for α-tubulin. Reports of tubulin immunoreactivity of human hepatic foregut epithelial cells are limited,^3,18^ and the expected morphology is not well defined. One study, using a pan–anti-tubulin antibody, illustrated foci of dense cilia in a single high-magnification image, however the grayscale image hinders detailed assessment.^3^ In our case, the immunofluorescence for α-tubulin highlighted cilia arranged in low-density aggregates, or as individualized cilia. Low-density cilia have been observed in ventral endoderm-derived tissues, such as the trachea, in neonatal mice.^4^ The tracheal cilia then increase in density during the postnatal period; therefore, the cilial morphology in our case could suggest that the ciliated cells are developmentally immature.

The liver adjacent to the foregut remnant had distorted lobular architecture, with randomly distributed arterioles and variable loss of portal structures. These changes are suggestive of concurrent microvascular dysplasia; however, given that macroscopically normal liver was not sampled, the extent of the dysplasia is not known. Reported cases of gallbladder agenesis, without communicating foregut cysts, had concurrent ductal plate malformations,^15^ and a single case had bile stasis and cholangiohepatitis.^9^ In our case, the resolution of clinical signs following surgery, lack of persistent biochemical abnormalities, and normal growth are suggestive that there had not yet been a clinically detectable impact on hepatic function.

Our case of a hepatic ciliated foregut remnant resulting in a direct communication between the common bile duct and the umbilicus, and concurrent gallbladder agenesis, caused bile to discharge from the umbilicus. Biliary sinus tracts or fistulas in any external location are rarely reported in the veterinary literature,^10,13,16^ and the involvement of a foregut remnant in our case is unique. Even within the human literature, direct communication of hepatic foregut cysts with the biliary tree in humans is rare.^17^ In humans, ciliated hepatic foregut cysts are more common in males.^8^ Our case was an immature male French Bulldog; the 2 reported cases of umbilicobiliary fistula in the veterinary literature were also in male dogs: a 1-y-old French Bulldog and a 1-y-old English Bulldog.^13,16^ The similarity in breed of these veterinary cases is notable. Other salient features of the published cases include concurrent gallbladder agenesis, but no involvement of the liver parenchyma in the French Bulldog, and in the English Bull dog, a functional gallbladder and a connection between the fistula and the right lateral liver lobe. The fistulas in both cases were identified histologically as aberrant bile ducts. Biliary fistulas in older dogs, or fistulas discharging to other cutaneous locations, have been reported, and were speculated to have potentially congenital or acquired etiologies.^10,12,14^

Human cases of hepatic foregut cysts are generally asymptomatic and are often found incidentally on imaging or during surgery, and are often managed conservatively.^11^ There are rare reports of malignant transformation of the tract epithelium into squamous cell carcinoma of the liver^21^; therefore, surgical resection in people has been suggested as the preferred management strategy.^8^

## Supplemental Material

sj-pdf-1-vdi-10.1177_10406387221147317 – Supplemental material for Patent hepatic ciliated foregut remnant resulting in an umbilicobiliary sinus tract, with gallbladder agenesis, in an 8-wk-old male French Bulldog Click here for additional data file. Supplemental material, sj-pdf-1-vdi-10.1177_10406387221147317 for Patent hepatic ciliated foregut remnant resulting in an umbilicobiliary sinus tract, with gallbladder agenesis, in an 8-wk-old male French Bulldog by Hannah E. Wong, John M. Cullen, Marta Vilà-González, Rachel Pittaway, Valeer J. Desmet and Tammy D. Gillian in Journal of Veterinary Diagnostic Investigation
