# Supplementary material for: Patent hepatic ciliated foregut remnant resulting in an umbilicobiliary sinus tract, with gallbladder agenesis, in an 8-wk-old male French Bulldog
Source: J Vet Diagn Invest. 2023 Jan 4;35(2):196–200. doi: 10.1177/10406387221147317 (PMC9999390; doi:10.1177/10406387221147317)
Supplement: sj-pdf-1-vdi-10.1177_10406387221147317 – Supplemental material for Patent hepatic ciliated foregut remnant resulting in an umbilicobiliary sinus tract, with gallbladder agenesis, in an 8-wk-old male French Bulldog [file sj-pdf-1-vdi-10.1177_10406387221147317.pdf]

JVDI: Supplemental material

Wong HE, et al. Patent hepatic ciliated foregut remnant resulting in an umbilicobiliary sinus tract, with gallbladder agenesis, in an 8-wk-old male French Bulldog

Immunofluorescence materials and method

Deparaffinization and rehydration of the mounted sections were followed by standard immunohistochemistry procedures. Antigen retrieval was performed with either citrate buffer (pH 6.0) or EDTA (1 mM, pH 8). Specimens were blocked with 10% donkey serum (Bio-Rad) and 0.1% Triton X-100 (MilliporeSigma) in PBS for 1 h at room temperature. Next, samples were stained with primary antibodies (goat anti-HNF3b AF2400, R&D, 3 μg/mL; mouse anti-acetylated tubulin 5335, Cell Signaling Technology, 1:800) diluted in 1% donkey serum and 0.1% TritonX-100 in PBS overnight at 4°C. After three 5-min washes with 0.1% TritonX-100 PBS, samples were stained with secondary antibodies (anti-rabbit AF568 and anti-goat AF488, Life Technologies, 1:1,000) for 1 h at room temperature. After two 5-min washes, nuclei were stained with 4′,6-diamidino-2-phenylindole (DAPI; 1:10,000) followed by 2 more washes with PBS only. Samples were mounted (Fluoromount-G mounting medium; Life Technologies), and images were taken (LSM 700 confocal microscope; Zeiss).

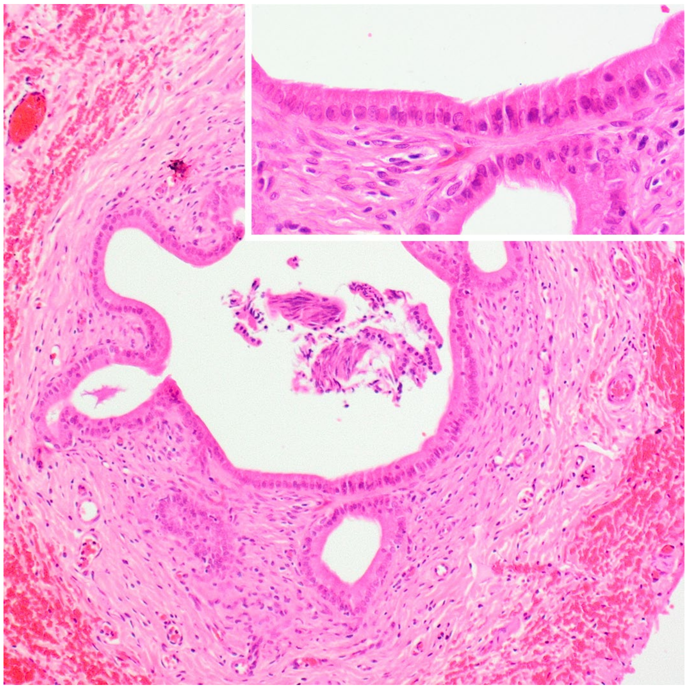

Supplemental Figure 1. Hepatic foregut remnant in the liver of a dog. The tubular structure is lined by ciliated columnar epithelium supported by a loose lamina propria and a thin, incomplete, smooth muscle layer, surrounded by a fibrous capsule. Low numbers of neutrophils, lymphocytes, and plasma cells are in the lamina propria. H&E. Inset: higher magnification of the ciliated epithelium. H&E.

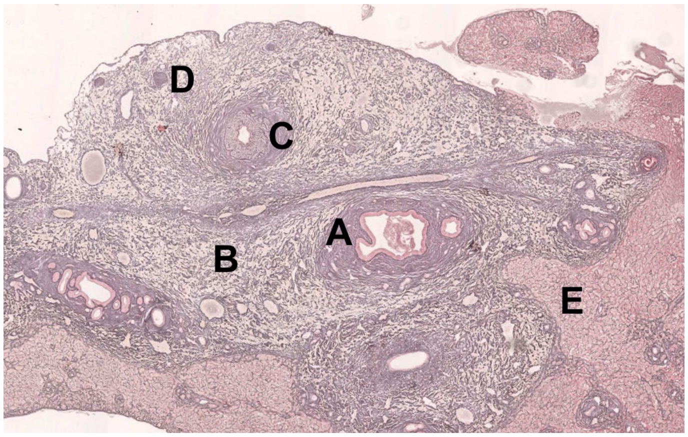

Supplemental Figure 2. Hepatic foregut remnant in the liver of a dog. Perihepatic sinus tract (A) is supported by abundant reticulin fibers (B), and accompanied by large vessels (C) and nerves (D), adjacent to recognizable hepatic parenchyma (E). Reticulin.

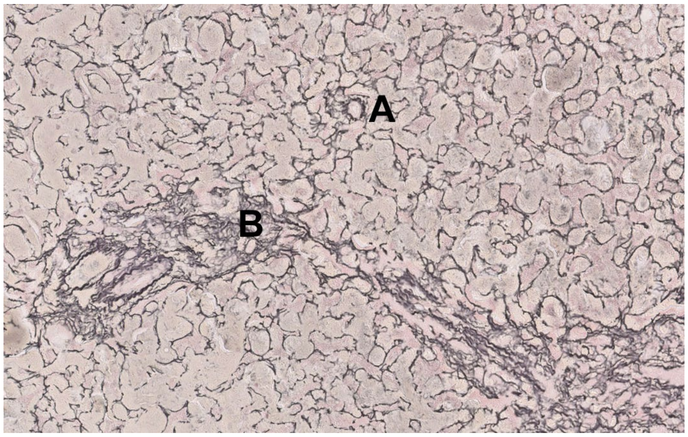

Supplemental Figure 3. Hepatic foregut remnant in the liver of a dog. Rare, isolated arteriole (A) within the parenchyma. B = portal area. Reticulin.
